# Thinking about internal states, a qualitative investigation into metacognitions in women with eating disorders

**DOI:** 10.1186/2050-2974-1-22

**Published:** 2013-07-04

**Authors:** Alix Vann, Esben Strodl, Erin Anderson

**Affiliations:** 1 Queensland University of Technology School of Psychology & Counselling, Level 5, O Block, B Wing, QUT Kelvin Grove Campus, Victoria Park Road, Kelvin Grove Queensland 4059, Brisbane, Australia; 2 Eating Disorders Outreach Service, 14 Cartwright Street, Herston Queensland 4029, Brisbane, Australia

**Keywords:** Eating disorders, Meta cognitions, Qualitative

## Abstract

**Background:**

There is a need for qualitative research to help develop case conceptualisations to guide the development of Metacognitive Therapy interventions for Eating Disorders.

**Method:**

A qualitative study informed by grounded theory methodology was conducted involving open-ended interviews with 27 women aged 18–55 years, who were seeking or receiving treatment for a diagnosed ED.

**Results:**

The categories identified in this study appeared to be consistent with a metacognitive model including constructs of a Cognitive Attentional Syndrome and metacognitive beliefs. These categories appear to be transdiagnostic, and the interaction between the categories is proposed to explain the maintenance of EDs.

**Conclusions:**

The transdiagnostic model proposed may be useful to guide the development of future metacognitive therapy interventions for EDs with the hope that this will lead to improved outcomes for individuals with EDs.

## Background

Given that eating disorders (EDs) are prevalent among women in western countries [1,2], there is an obvious need to develop effective interventions for eating disorders. Psychological therapies have been recommended as important components of interventions for EDs, with cognitive behaviour therapy (CBT) being a commonly recommended treatment [3,4]. While CBT has been helpful for many people, there are still limitations in its effectiveness. For example, meta-analyses have found that only approximately 41% of individuals with Bulimia Nervosa (BN) completing individual CBT interventions recover and only 29% remain recovered 12 months following CBT [5].

Given the variable and often limited impact of diagnosis-specific treatments for EDs, as well a recognition of additional maintaining mechanisms beyond the common cognitive targets of overvaluation and dieting, an enhanced cognitive behaviour therapy (CBT-E) was developed for EDs by Fairburn and colleagues [6,7]. This enhanced therapy was based on a proposed transdiagnostic cognitive model of EDs as well as the inclusion of four additional maintaining mechanisms: perfectionism, core low self-esteem, mood intolerance, and interpersonal difficulties. While Christopher Fairburn’s CBT-E has significantly improved the conceptualisation of eating disorders, trials using CBT-E indicate that there is still a need to further refine the case formulations and interventions for EDs. For example, in a sample of 154 patients with an EDs other than Anorexia Nervosa (AN), after receiving 20 weeks of CBT-E only approximately 50% of the sample had a level of eating disorder features less than one standard deviation above the community mean [7].

While such outcomes are still admirable given the complexity and pathology associated with EDs, these findings have resulted in calls by clinicians and researchers to further develop the cognitive models that underpin interventions for EDs [8,9]. One proposition has been to extend the standard cognitive therapy conceptualisation of focusing on the content of cognitions, or *what* people think, to *how* people think [10,11]. The domain of ‘how’ thinking occurs comprises the concept of metacognition, which refers to the cognitive mechanisms involved in the knowledge, interpretation and regulation of thinking itself [10]. One metacognitive theory has stemmed from the Self-Regulatory Executive Function (S-REF) theory of psychological disorder proposed by Adrian Wells [12]. The S-REF model suggests that beliefs comprise two metacognitive components that guide information processing, interpretations and the control of thoughts. The first is metacognitive knowledge, or the positive and negative beliefs which an individual holds about how to regulate internal states. The second is metacognitive regulation, or the coping strategies and changes in focus of attention that result from the chosen means of internal regulation [12]. S-REF theory suggests that in the case of psychological disorder, this metacognitive system becomes dysfunctional and the metacognitive processes become maladaptive [12], resulting in a Cognitive-Attentional Syndrome (CAS) [11,12]. The CAS involves perseverative thinking and excessive conceptual processing, as well as attentional hypervigilance to threat, which lead to the use of unhelpful coping strategies that ultimately fail [10,12].

Metacognitive theory has previously been applied in the conceptualisation and treatment of anxiety and depression with success [12,13]. However, the application to EDs has been limited thus far, with integrated cognitive and metacognitive therapy used only with BN [8]. This is perhaps due, in part, to the current lack of a sound metacognitive model of EDs that specifies which metacognitions underlie EDs, and are therefore important to target. The limited application of metacognitive theory in EDs may also reflect the reliance on deductive research methodologies by employing quantitative measures of metacognition that were developed for other psychological disorders [14,15]. Further, to date metacognitive research in EDs has focused on a single ED diagnosis [14], which is not consistent with emerging transdiagnostic conceptualisations of EDs [6]. As such, the lack of inductive investigations into metacognition in EDs may have restricted the depth of understanding of these disorders and the development of new metacognitive interventions.

Beginning to address these limitations, Woolrich and colleagues [16] recently investigated metacognition in patients with AN, dieters and non-dieting controls, using a qualitative methodology and found evidence for specific metacognitions associated with AN. Whilst providing a starting point for the current inductive research, the study by Woolrich and colleagues was limited by a number of methodological issues. Woolrich and associates [16] extracted data using a simple thematic analysis rather than any interpretative analysis, which would allow for the development of a model to guide case formulation. Further, the study explored metacognitions in a single diagnostic category (i.e. AN), not allowing for any comparison of metacognitions across ED diagnoses. Thus despite preliminary investigation into the existence of metacognition in EDs, there is still no in-depth qualitative investigation of metacognition in EDs available. Another limitation we see in the research is that some metacognitive theories focus solely on metacognitions about verbal cognitions [10-12]. Recently there has been a call to build upon metacognitive theory by also considering meta-cognitions about emotions [17]. Using Well’s metacognitive theory as a basis, Manser, Cooper, and Trefusis developed a questionnaire measuring metacognitions about emotions and found that metacogntions of emotions added to the explanation of emotional dysregulation (as measured by borderline personality disorder symptoms) above that explained by metacognitions about verbal cognitions [17].

The current research therefore aimed to propose a theoretical model to guide the conceptualisation of metacognitions in EDs that might guide the development of future metacognitive interventions for EDs. As such given the presence of existing metacognitive therapy strategies and interventions, based upon Wells’ SREF model [11], starting questions for the exploration were based upon Wells’ SREF model. In addition the study aimed to expand this model by also exploring metacognitions about emotions as well as metacognitions about verbal thoughts. Finally given the emerging evidence of transdiagnostic conceptualisations of EDs, we were also interested to explore if categories of metacognitions varied across diagnosis or tended to be transdiagnostic. A grounded theory approach [18] was chosen as a basis to provide a rigorous framework that would support not just the extraction of themes but also an interpretative analysis for the development of a model. Due to the higher prevalence of EDs in females and in order to keep the sample relatively homogenous, the current research sampled women only.

## Method

### Participants

Participants were 27 women between the ages of 18 and 55 years, with the majority of participants 30 years old or younger (mean = 26.22 years, *SD* = 8.11 years). Participants were attending the Eating Disorders Outreach Service (EDOS) at a major metropolitan hospital in Brisbane, Australia, and were seeking or receiving treatment for an ED (AN, BN or EDNOS). Participants satisfied criteria for an ED diagnosis according to the DSM-IV-TR^a^[19] and received a diagnosis from a psychiatrist or a psychiatric registrar as part of the intake process at EDOS, which was confirmed by administering the ED section of the Mini International Neuropsychiatric Interview [M.I.N.I.] 6.0. All patients who engaged with EDOS during the data collection period were considered for participation. EDOS clinicians (clinical psychologists, psychiatrists or psychiatric registrars) individually assessed the suitability of patients to participate based on current mental state and overall functioning. No specific exclusionary criteria were applied.

### Setting and procedure

Data collection was approved by the hospital and university Human Research Ethics Committees. Interviews were conducted between April 2011 and May 2012 and were audio recorded. Each individual interview lasted between 20 and 60 minutes. Following the interview, participants were administered sections L. Anorexia Nervosa and M. Bulimia Nervosa of the M.I.N.I. 6.0 by the first author (AV). In addition, AV returned to each section of the M.I.N.I. and asked appropriate questions to ensure the diagnosis was congruent with proposed DSM-V diagnoses. Participants were finally asked to complete a brief demographic questionnaire regarding their age, culture, educational background/qualifications, time since diagnosis, and number of hospitalisations related to the ED.

The initial starting questions were based upon Wells’ SREF model i.e. positive and negative metabeliefs, attentional bias and coping strategies i.e.

1 What do you think is good about experiencing strong emotions or thoughts?

2 What do you think is bad about experiencing strong emotions or thoughts?

3 When you feel that urge, what do you do to cope with experiencing strong emotions or thoughts?

4 What do you focus your attention on when you experience strong emotions or thoughts?

In line with grounded theory methodology, questions were modified as categories and a model emerged through the process of open and axial coding [18] with metacognitive profiling questions [10] were drawn upon as necessary to further elucidate the emerging themes.

### Data analysis

AV transcribed interviews verbatim. All coding was conducted from transcriptions and was done in line with grounded theory methodology using the ATLAS.ti v6.2 (2010) computer software. Open coding proceeded in a two stage process. The labels associated with the codes and concepts extracted from the transcripts were aligned closely with the words used by the participants. As the authors worked with the data generation, the categories appeared to overlap significantly with the constructs proposed by Adrian Well’s metacognitive model. As such words and terms were borrowed from this metacognitive model to more accurately describe the categories. As concepts and categories started to emerge from within the data, axial coding was used to relate concepts and categories to each other, with an emphasis on causal relationships, intervening conditions, action strategies and consequences [18]. In addition to the original interview questions posed, more specific questions were asked in following interviews to confirm, disconfirm and explore the emerging core categories and the proposed model. Finally, selective coding was used to relate all other themes, relationships, conditions and consequences to the core categories identified in the overall model. Once saturation of themes was reached in the data analysis, the concurrent data collection and analysis allowed for the final four interviews to be largely confirmatory/disconfirmatory in nature. In these interviews, participants were presented with an outline of the proposed overall model and asked specific questions to determine whether or not this was in line with their experience.

Theoretical memos were used throughout the data collection and analysis process to track working hypotheses and rationales for the process of coding, and aid the generation of a theory with well-connected concepts. Reflexivity was also employed to ensure that the researcher’s personal beliefs and biases, and their potential influence on the analysis, were considered. The first author was a postgraduate student who had been briefly trained in psychodynamic therapies, cognitive behaviour therapy, narrative therapy and family therapy, with cursory prior knowledge of metacognitive therapy. She had no strong allegiance to any particular modality of therapy. The second author is an experienced clinician who works primarily from a cognitive behaviour therapy framework and had a relatively strong prior knowledge of metacognitive therapy principles. The third author is an experienced clinician who works primarily from a cognitive behaviour therapy framework and had no prior knowledge of metacognitive therapy. To further address methodological rigour, the second and third authors reviewed interview transcripts in order to confirm the validity and integrity of coding.

## Results

### Demographic summary

The breakdown of diagnoses of those interviewed was: nine AN, nine BN, and nine EDNOS. All participants were Australian-Caucasian except for one American-Caucasian and one Asian participant. The mean body mass index (BMI: weight [kg]/height [m^2^]) at the time of the interview per diagnostic group was as follows: AN mean = 17.22 (*SD* = 1.22); BN mean = 22.79 (*SD* = 1.84); EDNOS mean = 20.44 (*SD* = 2.68). The length of time since initial diagnosis ranged from four months to 20 years, and 21 of the participants had been diagnosed with an ED for over a year. Eleven participants had been hospitalised at some point since diagnosis, due to the ED.

### Overall model summary

**Figure 1:**
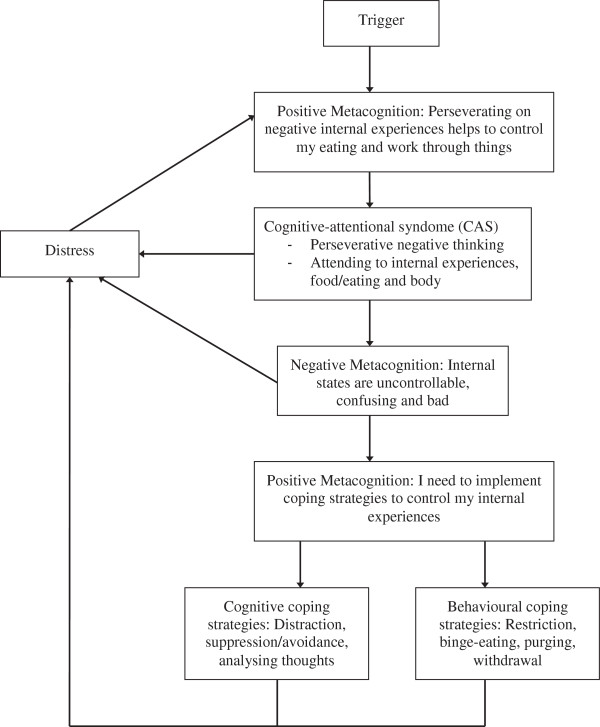
Proposed model of metacognition in EDs.

The results are presented within the structure of the model that emerged from the ongoing data analysis. Comparison of the themes across diagnostic categories supported a transdiagnostic model of metacognitions in EDs and is displayed in Figure 1 below. Axial coding revealed that the sequence of actions within the model started with specific, but not universal, triggers activating participants’ positive metacognitive beliefs about internal states (namely negative thinking and strong negative emotions). Once these beliefs were activated, participants would employ the perseverative negative thinking and specific allocation of attention found to be characteristic of the CAS in EDs. It appeared that the CAS itself resulted in some distress for participants. Once the CAS was active, participants’ negative metacognitive beliefs about internal states were then activated, with these beliefs adding to the distress participants experienced. Following this, positive beliefs participants held about their coping strategies arose, which resulted in the implementation of several specific cognitive and behavioural coping strategies. Whilst providing some temporary relief, these strategies ultimately increased the experience of distress. Given that distress in itself is an internal state, the experience of distress then had the potential to re-activate positive beliefs about internal states, thus re-activating the original cycle. These themes and actions are described in more detail below.

### Metacognitive model for eating disorders

### Trigger

All participants idicated that there was usually an external trigger that began the process of activating their metacognitive beliefs. This was often related to body image, weight, food or eating, or as described below, may also have been related to more general events:

“*Well it usually starts off with*, *it usually happens*… *after I*’*ve put on weight or I look in the mirror and I see something that upsets me and then it sort of progresses to*… *a want to be incredibly skinny again*… *I mean it could be anything from eating an extra apple to someone making a comment*, *like*, *completely unrelated to body shape and* [*the*] *eating disorder*.” (Age 18 – BN)

Whilst this trigger was usually external, for the majority of participants it seemed to result in an internal trigger, in the form of either a distressing thought or a strong negative emotion. The internal trigger seemed to lead to the activation of positive beliefs held about certain ways of regulating internal experiences.

### Positive metacognitions about internal states

The data revealed three dominant beliefs about positive actions to help regulate the experience of internal states. With the first two metabeliefs below, in both cases the perceived positive action is the same (i.e. engaging in negative perseverative thinking), however in the first case this is due to interpreting emotions as uncontrollable and dangerous, whereas in the second case it is due to interpreting thoughts as uncontrollable and dangerous.

1. Strong negative emotions mean something uncontrollable is happening and therefore I need to focus and perseverate on my negative thoughts to protect me from losing control of my eating, because if I lose control of my eating I will become fat and worthless.

This category was endorsed by most participants. In the quote below, an emotion triggers off the action of perseverative negative thinking as a strategy for protection against losing control of one’s eating behaviour.

“*[I focus on] not eating*. *I don*’*t know when that started but when I*’*m angry*, *when I*’*m sad*, *like I*’*m a bit sad today*, *like a bit off*, *and the next step is* [*to think*] *don*’*t go to the fridge*, *don*’*t you dare go into the kitchen*, *do not eat*, *just do not blow it*, *like you feel shit*, *there*’*s an opportunity that you*’*ll just lose control*, *like do not do that*.” (Age 26 – EDNOS)

2. Negative thoughts mean something uncontrollable is happening and therefore I need to focus and perseverate on my negative thoughts to protect me from losing control of my eating, because if I lose control of my eating I will become fat and worthless.

The quote below illustrates an emergent theme that engaging in negative perseverative thinking was a positive action, in response to experiencing negative thoughts, that resulted in benefits for the person by protecting them from losing control of their eating or protecting them from becoming fat and therefore worthless. The majority of participants endorsed this category.

“*If I didn*’*t think those*, *I suppose if I didn*’*t think that stuff then I might just actually eat all day every day*… *you know just eat all the time um and then I would become more enormous*… *Yeah if I beat myself enough with the negative thoughts and stuff somehow that might make me a better person*… *And certainly*, *oh I would certainly be a much better person if I were smaller*.” (Age 55 – EDNOS)

3. Feeling strong negative emotions and thinking negatively is comforting as it is familiar and means that I am working hard at something, therefore it is good for me to focus on my negative internal experiences.

Participants indicated that the experience of strong negative emotions and negative thoughts was often painful, uncomfortable and overwhelming. This category was endorsed by approximately half of the sample. However, paradoxically, the experience of the emotion or thought was still viewed as positive action in regulating their internal state in that it allowed them to feel comforted and reassured by the familiarity of the experiences:

“*It gives me something to always think about*… *because it*’*s become part of me*, *it*’*s like*, *if I didn*’*t have it then what*’*s wrong*?… *What am I supposed to think about all the time*?” (Age 29 – AN)

There was also the sense for participants that experiencing perseverative negative thinking allowed them to acknowledge or work through something, so focusing on their negative thoughts may lead to something positive:

“*The negative self*-*talk may have in a way made me go ok you*’*ve screwed up*, *you didn*’*t do that right*… *Yeah it*’*s good because you feel real and you feel like you*’*re making progress*, *but it*’*s hard when you*’*re in it*.” (Age 28 – BN)

### Cognitive-Attentional Syndrome (CAS)

In line with previous metacognitive models of psychopathology, it appeared that the majority of women with EDs in the current study had a CAS that was activated at this point. Two aspects of this which appeared to be consistently endorsed by participants included the implementation of perseverative negative thinking and attentional bias.

### Perseverative negative thinking

All participants employed perseverative negative thinking in some form. The perseverative thinking described by the participants included worry about the future and rumination about the past, but was predominated by strong themes of repetitive thoughts of food/eating/body image. The quote below is an example of a perseverative negative thinking pattern of one participant.

“*I think*, *no*, *don*’*t think about that*, *don*’*t analyse the thoughts just do what you used to do because that made you happy once*… *and I keep telling myself that restricting is good and that getting on the scales and weighing less every week is good*… *but I*’*ll sort of think at the same time that*’*s not good to think that way because is it actually true*? *Like lately I weigh myself and I don*’*t feel happy anymore and then I think what*’*s wrong with you*, *you used to love getting on the scales and being so thin*. *But now I just think*, *why do you think that*… *why aren*’*t you happy*?” (Age 22 – AN)

### Attentional bias

There was a consistent sense for participants that when they were experiencing strong negative emotions or negative thoughts, their attentional focus would become largely internal, such that these experiences caused them to focus on their thoughts (often their negative, ED-related thoughts):

“*I should be going out and doing things*, *well I do go out but then I go out and it*’*s like oh*, *you*’*re away from the fridge*, *that*’*s good*, *that*’*s good*, *but then [I*’*m thinking] when you get home don*’*t eat*, *make sure when you get home you don*’*t eat*… *so a big focus on thoughts of not eating*.” (Age 26 – EDNOS)

Participants also reported an increased focus on food and/or eating when they were experiencing a strong negative emotion or negative thoughts:

“*I guess [my focus was] on the actual food that I was eating like just*, *like if I just restricted what I was eating well then I wouldn*’*t have to be worried so much that I was over*-*eating or that I would gain weight or that kind of thing*.” (Age 21 – BN)

When in a state of heightened emotion or engaging in negative thinking, it was also common for participants to attend more closely to their bodies, their weight and how they compared to others on these dimensions:

“*I think because I was so body focused*, *mentally as well*, *I really experienced emotions quite physically and I would*, *more so than I had in the past like you know feel things here and here* [pointing to back and tummy] *and just that drained sadness*… *I remember being really aware of what was sore and what was*, *you know like you*’*d be kind of very body*-*focused*.” (Age 22 – BN)

While most participants endorsed an attentional bias towards thoughts and body image/weight, only some participants reported attentional bias towards food.

### Negative metacognitions about internal states

With the CAS activated, all participants described negative metacognitive beliefs related to the regulation of internal states. The negative metacognitive beliefs reported by participants are best represented by the following three categories:

### Strong emotions and negative thoughts are uncontrollable and awful

Participants consistently described the sense that their experience of strong negative emotions and negative thoughts was overwhelming and all-consuming, such that they felt powerless to be able to do anything about them or to stop the emotions/thoughts once they had started. This was a category that was endorsed by almost every participant.

“*The symptoms of the emotion that I was feeling*, *like*, *I had no control of it whatsoever and it was the first time that I ever felt that intensely*, *like where I couldn*’*t stop crying*… *I just couldn*’*t turn it off*, *I didn*’*t know how to turn it off*.” (Age 28 – EDNOS)

Participants described their strong emotions and negative thoughts as simply awful, and experiencing these internal states as terrible, uncomfortable, painful and exhausting:

“*Emotions for me*, *like feeling emotions means like I*’*m just hurt*, *I get hurt*, *or it*’*s painful*… *anger for me is*, *it*’*s just a painful experience*, *like if you slammed your hand in a car door sort of pain*, *it just hurts to be angry*.” (Age 26 – EDNOS)

### Strong emotions are confusing – I don’t know what I am feeling or what to do about the feeling

Most participants described having difficulty identifying the emotion they were feeling, struggling to label or describe the emotion, and finding it difficult to approach strong negative emotions in a rational sense:

“*Yeah I think as well like*, *with the feeling*, *a lot of it was suppressed for so long that*’*s why it*’*s really hard to tell*, *you know*, *what are you feeling*? *Cos I*’*m quite*, *I*’*m extremely emotional and I really hate it but*… *I actually am not really good at picking up*, *like different types of emotions* … *and you know like in one of the therapy sessions she was like* ‘*and how did that make you feel*?’ *and I was just like* ‘*I dunno*’, *like*, *to label it I find strange*.” (Age 29 – BN)

From this confusion, these participants also appeared to struggle to know what to do with strong negative emotions, grappling with how strong emotions should be handled and whether there was anything that could be done about them:

“*I actually had a very strong desire to cut myself um because I was unbelievably angry slash frustrated*, *I suppose the feeling is anger*, *and I couldn*’*t think of anything to do and I actually had the conscious thought well what is an appropriate way to react to that*?” (Age 55 – EDNOS)

### All negative thoughts are bad, wrong or abnormal and need to be addressed

Approximately half of the participants frequently passed judgements on their thoughts and their thinking, labelling many of their internal experiences as bad, wrong, unjustified or abnormal:

“*There isn*’*t really anything good about it* [negative thinking]… *it*’*s not something I should feel or think*, *it*’*s bad*, *but I do*… *yeah and then I think that I*’*m a terrible person because I don*’*t think normally like you know*, *other normal healthy people do*.” (Age 22 – AN)

These participants also appeared to hold strong beliefs about the need to address negative thoughts each time they occurred. It seemed important that they did not simply accept their negative thoughts as they occurred, but that participants worked hard to challenge, control or change their negative thoughts:

“*If I*’*m aware of my thoughts and I just challenge them as they come*, *and if I hear a bad thought*, *I will tell myself what I need to be thinking to replace that thought*.” (Age 23 – BN)

### Positive beliefs about coping strategies

Similar to the positive beliefs identified about internal states, more than half of the participants also appeared to hold positive beliefs about the coping strategies they employed to manage their experience of internal states. Such beliefs appeared to be activated after the negative metacognitions and prior to the implementation of their coping strategies. Positive metacognitions about coping strategies were initially explored as they related to managing thoughts and emotions separately, however, as the analysis continued, the data suggested that the types of beliefs held by participants were similar for both of these types of internal experience. As such, the dominant positive belief appeared to be: I need to implement my coping strategies intensely to protect me from being taken over by my internal experiences (e.g., thoughts and emotions) because if I lose control of my internal experiences, I will lose control of my eating and become fat and worthless.

### Cognitive coping strategies

Following these beliefs, several cognitive coping strategies were consistently identified by participants as being employed to reduce the distress experienced to this point.

### Distraction

Most participants described using cognitive distraction to ensure that their mind was otherwise engaged or busy. Participants appeared to employ this strategy to escape or manage the experience of strong negative emotions or difficult thoughts:

“*I*’*d say even though distracting myself is hard to do*, *I tend to find that a bit more effective in terms of forgetting about the negative thoughts*. *Even though it might be hard to actually start a crossword puzzle or to you know make the effort to go and see someone*, *once I actually do then I tend to you know feel better afterwards*, *yeah*, *or less stressed about everything*, *even though it*’*s harder to actually do*.” (Age 22 – AN)

### Suppression/avoidance

Most participants also reported trying to mentally block out or avoid some negative thoughts and/or strong emotions completely. For some participants this was done as a way of numbing their minds to avoid reality:

“*You disconnect from all emotion I think sometimes*… *and that includes all the positive ones that disconnection*, *you reject love and things like that as well so you have to*, *in order to get rid of the bad stuff you have to sacrifice all of the good things*, *yeah you can*’*t*… *um block out the sadness and keep the love* – *either it all goes or it all stays*.” (Age 22 – BN)

### Analysing/questioning negative thoughts

There also emerged a strong belief amongst participants that it was important to cognitively address their negative thoughts and strong emotions in some way, and that doing so would help them to cope. The data showed that the idea of “thinking things through” was important and this appeared to encompass trying to analyse thoughts and feelings, questioning and challenging negative thoughts, and trying to control negative thinking:

“*When I was actually out of control was when I was in control*, *and I think a lot of that would have come from that weighing up and deciding is it good*, *is it not*, *should I do it*, *should I not*, *and every decision was calculated almost*, *even if it was subconscious*, *it was always a calculated thing*… *it was always controlled*.” (Age 22 – EDNOS)

This category was endorsed by approximately half of the sample.

### Behavioural coping strategies

Also following from the positive beliefs about coping strategies, several behavioural strategies were employed by participants to cope with their distress.

While there was a general tendency for some diagnostic disorders to use particular behavioural coping strategies more than other diagnoses (e.g., restricting food intake/starvation was typical of participants with AN, while binge-eating was typical of participants with BN), the striking observation was that the underlying purpose for a strategy selection was transdiagnostic.

#### Restricting food intake/starvation

Participants with AN described using restriction of their food intake as a way of controlling strong negative emotions and perseverative negative thinking. This was often expressed in terms of the “numbing” of the mind that successful restriction allowed, perhaps due to the physiological response of the brain undergoing Starvation Syndrome. In this way, this behavioural coping mechanism may partly have been employed as another way of maintaining cognitive control.

“*For me it*’*s like a numbing process*. *Once my brain*’*s starved and I*’*m fairly numb*, *you can*’*t think about much at all*.” (Age 24 – AN)

Similarly impacting cognitive functioning, restriction appeared to be employed as a means of consuming the mind with thoughts of food and eating, to avoid thinking about other, more distressing, thoughts:

“[*I would*] *try to concentrate a lot because you know I*’*m allowed three milkos* [biscuits] *a day*, *and you know having five milkos in my bag and then having to concentrate and tell myself even though I*’*m really really hungry that I can only have three and it*’*s really really hard sometimes to not want to have the fourth so then it takes all my energy and all my focus and all my concentration to be worrying about not having those other two milkos and so then I don*’*t have to think about*, *you know*, *my day*.” (Age 39 – AN)

Approximately half of the sample reported using this coping strategy irrespective of the diagnosis.

### 2. Binge-eating

Across diagnoses, most participants reported binge-eating food as a coping mechanism. Several purposes to this strategy emerged from the data, including using the episode of binge-eating as a distraction or a means of escapism. For some women bingeing appeared to result from building feelings of being out of control. These women initially restricted their food intake in response to feeling out of control, but once the feeling reached a certain threshold, then they felt that they *were* out of control. At this point, feeling like they had to act on their thoughts of being out of control, some women engaged in the disinhibited eating that is characteristic of binge-eating:

“*I felt really sad and out of control*… *so I just was like what*’*s the point*? *I*’*m just going to give in and have that food suicide*… *I don*’*t think I really thought past the point I*’*m just going to eat all this food and become a big slug and I don*’*t know what would happen then I guess I*’*d be worthless and non*-*existent and things wouldn*’*t matter and I*’*d just sort of die*.” (Age 19 – AN)

Binge-eating also seemed to be employed due to the experience of positive cognitive effects during bingeing. Some participants reported that food and eating became their sole focus in this moment, allowing their mind to feel cleared of any thoughts:

“*And I never realised that until recently but like whenever I would eat*, *it would be to the extent that I just would*, *I would zone out*, *it was just me and my food*. *And I wouldn*’*t*, *I just wouldn*’*t think*.” (Age 22 – EDNOS)

### Purging

Approximately half of the participants in each diagnostic group reported the use of various purging strategies to cope with strong negative emotions and difficult thoughts. Such strategies included vomiting, exercising, and the use of laxatives and/or diuretics. Purging may have been somewhat of a secondary coping mechanism designed to deal with the added distress caused by the use of the previous two coping strategies. However, purging does appear to act as a form of release to cope with a build up of internal pressure, in particular feelings of guilt:

“*I end up purging because it*’*s like I need to feel some sense of relief or I need to take the feeling of guilt associated with purging to remove my mind from the intensity of* [*it*].” (Age 28 – EDNOS)

### Social withdrawal

Most participants frequently used withdrawal from others as a way of dealing with their strong emotions and/or negative thoughts, regardless of diagnosis. Participants reported that social isolation helped them to control their experiences by allowing them to deal with things alone:

“*It did take me a few years to kind of get to a place where I was isolated*… *but when I got there I was like this is my world and like you can come and walk around me and you can say things to me but they*’*re going to bounce straight back off me because I kind of formed like a whole new identity within myself*… *and like drawing from the eating disorder*, *that was my only friend I guess*, *that was the only thing that I would let in my life and so once I got to that place I was like* ‘*Hi*, *I*’*m great*, *life is great*, *because you*’*re not in it*!’” (Age 27 – BN)

## Discussion

Grounded theory guided the combined inductive/deductive interpretative analysis to produce a metacognitive model which may guide the future development of metacognitive interventions for EDs. We found evidence that women with EDs do experience a Cognitive-Attentional Syndrome (CAS) that involves perseverative thinking and attentional bias about food/body image/weight. Consistent with metacognitive model proposed by Adrian Wells [12], we found evidence that women with EDs do have positive and negative metacognitions surrounding the CAS that appear to drive both the experience of the CAS and the selection of unhelpful coping strategies to manage the CAS. While the experiencec of the CAS and coping strategies help to define the manifestation of the ED, the associated metacognitions and attentional bias are proposed also to be causal factors in this presentation and should be considered targets for interventions. The model proposed was transdiagnostic in nature, as the qualitative analysis suggested no clear differences between diagnoses in the existence, or degree of endorsement, of the core categories.

The overarching categories are in line with previous models of metacognition in psychopathology. Similar models have been proposed for generalised anxiety disorder (GAD) [20], obsessive-compulsive disorder [21], post-traumatic stress disorder (PTSD) [22], and depression [23]. In the current study, some of the beliefs identified about negative thinking are similar to the beliefs about worry proposed as important in GAD (e.g., my worries are uncontrollable) and to the beliefs about rumination suggested to be key in understanding depression (e.g., thinking about the causes of depression will help me to prevent it). The results of the present research thus support the application of metacognitive theory to EDs, and suggest that there is some overlap between important metacognitive constructs in EDs and other forms of psychopathology that may help explain the high levels of comorbidity seen in these presentations.

### Metacognition about emotions

In line with recent suggestions about the importance of metacognitions about emotions [12], we found support for the role of metacognitions of emotions in EDs. In particular we found themes of beliefs that emotions can be uncontrollable or dangerous, or interestingly also confusing. The concern that emotions can be uncontrollable or dangerous is consistent with Wells’ identification of metacognitions that verbal thoughts can be uncontrollable or dangerous [12]. The theme of emotions being confusing is consistent with findings of higher levels of alexithymia in those with EDs [24-27].

Within the construct of alexithymia, the affective elements (e.g., problems identifying and expressing emotional states) has been suggested as more relevant to women with EDs than the cognitive elements (e.g., poor externally oriented thinking) [24]. The current study supports this literature by suggesting that one of the reasons for employing ED behaviours may be to manage the distress associated with feeling confused by strong negative emotions.

### Transdiagnostic considerations

An important consideration in this research was the suitability of adopting a transdiagnostic perspective of EDs. The issue of whether metacognitive beliefs and related constructs were transdiagnostic was considered throughout the qualitative analysis process, particularly when undertaking axial and selective coding to relate themes to each other. In line with recent developments [5], the current research supports the view of EDs as transdiagnostic when considered within a metacognitive framework. There did not appear to be meaningful qualitative differences between the metacognitive beliefs, the CAS or the cognitive coping strategies employed by participants with AN, BN and EDNOS. While there were some broad differences in the selection of behavioural coping strategies, the underlying purpose for the implementation of these strategies appeared common across diagnostic groups. Further investigation into the transdiagnostic nature of metacognitions in EDs is recommended.

### Treatment implications

Given the variable impact of currently available treatments of EDs on recovery, there is a need to continue to explore new conceptualisations of EDs to develop interventions with improved outcomes. One option is to explore the impact of metacognitive therapy for the treatment of EDs. There is emerging evidence from other diagnostic categories such as PTSD [22], GAD [20,28] and depression [23,29] that metacognitive therapy may be a promising development over traditional CBT. The themes and model identified in this study may guide case formulation required to implement metacognitive therapy for eating disorders and spur further research in this area.

In particular, it is suggested that interventions should target the main themes extracted from this study. That is, clinicians and researchers should target positive metacognitions that motivate the use of perseverative negative thinking about eating, body image, weight etc. In addition, they should target the negative metacognitions that emotions and thoughts are dangerous or uncontrollable and that an individual needs to use a coping strategy to manage their emotions and thoughts when they experience them. We also feel that clinicians and researchers should use metacognitive strategies to address the attentional bias toward internal cues as well as towards cues of food, weight or body image. Moreover, standard metacognitive therapy strategies could be used to help clients or participants change their use of the unhelpful cognitive and behavioural coping strategies identified in this study. However in addition to using standard metacognitive therapy strategies for areas identified in this study, we believe that individuals with EDs would also benefit from further strategies aimed at helping them better understand their emotions. A common theme was that emotions were confusing with the implication that the participants therefore needed to use an unhelpful coping strategy to manage these confusing emotions. The addition of psychoeducation in affect regulation to treatment as usual (CBT) has been shown to improve the skill of down-regulating negative affect in patients with AN and BN, and to also reduce dietary restraint [30]. This suggests that educating individuals with EDs about emotions can improve their ability to handle emotions and impact their ED symptomatology. Other researchers who view EDs as an emotion regulation strategy have administered dialectical behaviour therapy (DBT) [31] to women with EDs. Modified DBT for EDs has been shown to be effective in addressing both ED behaviours and more general forms of psychopathology (e.g., mood disorders, anxiety) in ED samples [311]. However, the effect of DBT for EDs on the core element the treatment theoretically targets – affect tolerance and regulation – is less clear [32]. As such, it is possible that even treatments with a specific distress-tolerance component such as DBT are not directly impacting this ability in women with EDs. Thus, it is important to continue considering methods to directly target the affective difficulties connected with EDs. The current model provides another way of thinking about core emotional difficulties in EDs and may eventually be used to inform other potential treatment modalities, such as detached mindfulness as part of MCT.

### Limitations

All participants were recruited via their engagement with a specialist ED treatment service (EDOS) where they were offered or already engaged in CBT treatment. Engagement in structured CBT interventions may have impacted the metacognitions elicited during interviews and may be a limitation in the current study. This impact is particularly relevant to beliefs about the need to challenge/analyse thoughts, as this concept is fundamental to CBT treatment approaches. However, the idea of needing to be aware of, monitor and analyse one’s thoughts has been identified as a common cognitive coping strategy in other presentations of psychopathology [33]. It is therefore possible that the identification of the negative metacognitive belief that negative thoughts must be addressed and analysed may be a true metacognitive belief in women with EDs, rather than a belief shaped by treatment experiences. To confirm this aspect of the proposed model, future research should consider samples of women with EDs who have not yet undergone psychological treatment.

The current model does not clearly specify which strong negative emotions are implicated in metacognitive processes, or why ED symptoms are chosen as emotion regulation strategies instead of other behaviours. Such limitations have been previously criticised in cognitive-affective models proposed to understand EDs [34]. It was clear that participants held beliefs about negative, and not positive, strong emotions, and while they referred to a variety of strong negative emotions (including sadness, anger, guilt and fear) these were not specifically accounted for in the current model. The lack of specificity in exploration of emotions was due to the fact that no clear pattern emerged early on, and given the scope of the investigation, it was decided to keep this as a broad category to aid with conceptualisation. Future research should investigate whether particular strong negative emotions are implicated in a metacognitive model of EDs.

## Conclusion

To date, interventions targeting EDs have tended to only produce modest effects. It is possible that this may be due to a need for more novel conceptualisations of key psychological processes driving ED presentations. The findings from this study suggest that women with EDs do possess important metacognitions that may explain the psychopathology associated with EDs. In particular, we propose that the positive and negative metacognitions surrounding the CAS identified in this study are important targets for interventions. If the results of this qualitative study are replicated through deductive studies that confirm the generalisability of these findings, the themes identified in this study may provide important constructs to help develop case conceptualisations that guide future metacognitive therapy interventions for EDs.

## Endnotes

^a^Proposed DSM-5 criteria (as at April 2011, when data collection began) were also considered, and this altered the diagnostic breakdown accordingly: 10 AN, 8 BN, 1 Binge-eating Disorder, 3 Feeding and Eating Condition Not Elsewhere Classified (FECNEC) – atypical AN, 4 FECNEC – sub-threshold BN, and 1 FECNEC - other. However, this did not meaningfully impact the analysis and so DSM-IV-TR criteria were used for this study.

## Competing interests

The authors declare that they have no competing interests.

## Authors' contributions

AV conducted the data collection and performed the transcribing of the interviews. AV also contributed to the study design, data coding, the data interpretation, as well as the writing of the manuscript. ES contributed to the study design, data interpretation, as well as the writing of the manuscript. EA contributed to the study design, data interpretation, as well as the writing of the manuscript. All authors read and approved the final manuscript.
